# A bidirectional relationship between physical activity and executive function in older adults

**DOI:** 10.3389/fnhum.2014.01044

**Published:** 2015-01-13

**Authors:** Michael Daly, David McMinn, Julia L. Allan

**Affiliations:** ^1^Behavioural Science Centre, Stirling Management School, University of StirlingStirling, UK; ^2^School of Medicine and Dentistry, Rowett Institute of Nutrition and Health, University of AberdeenAberdeen, UK; ^3^Health Psychology, Institute of Applied Health Sciences, University of AberdeenAberdeen, UK

**Keywords:** physical activity, health behavior, executive function, cognitive ability, longitudinal design

## Abstract

Physically active lifestyles contribute to better executive function. However, it is unclear whether high levels of executive function lead people to be more active. This study uses a large sample and multi-wave data to identify whether a reciprocal association exists between physical activity and executive function. Participants were 4555 older adults tracked across four waves of the English Longitudinal Study of Aging. In each wave executive function was assessed using a verbal fluency test and a letter cancelation task and participants reported their physical activity levels. Fixed effects regressions showed that changes in executive function corresponded with changes in physical activity. In longitudinal multilevel models low levels of physical activity led to subsequent declines in executive function. Importantly, poor executive function predicted reductions in physical activity over time. This association was found to be over 50% larger in magnitude than the contribution of physical activity to changes in executive function. This is the first study to identify evidence for a robust bidirectional link between executive function and physical activity in a large sample of older adults tracked over time.

## Introduction

Advancements in medical science in the past century have markedly increased life expectancy but have also heralded a broad set of challenges that accompany an aging population (United Nations, 2011). Age-related cognitive decline is one such challenge, producing wide ranging psychological, social, and economic consequences at both the individual and population level (Frank et al., 2006; Olesen et al., 2012). Emerging evidence suggests that age-related neurocognitive-decline should not be seen as fixed or immutable (Hamer and Chida, 2009). Rather, cognitive function seems to benefit from a healthy lifestyle, most notably from regular physical activity (Hertzog et al., 2009; Ku et al., 2012; Gow, 2013).

This study examines whether engaging in physical activity attenuates declines in higher level cognitive function (executive functioning) over a period of 6 years in older English adults. In addition, the current study investigates the more novel prediction that the relationship between physical activity and cognitive performance is bidirectional, and that executive function will also play a *predictive* role in shaping activity levels over time (Batty et al., 2007; Sabia et al., 2010). The executive functions, in particular, may enable people to consistently engage in effortful behaviors like physical activity in order to achieve long-term health benefits (Hall and Fong, 2007). As the contribution of executive functioning to physical activity has not yet been established in large scale prospective studies, this study also aimed to test this pathway in a sample of more than four thousand older English adults.

### Physical activity as a determinant of executive functioning

The executive functions are higher level cognitive processes associated with the frontal lobe of the brain that control and co-ordinate more fundamental processes in the effortful pursuit of goals (Alvarez and Emory, 2006). Complex and multi-faceted in nature, the executive functions include planning, selective attention, sustained attention, the ability to deal flexibly with novel situations, and pre-potent response inhibition (Hall et al., 2008b; Hofmann et al., 2012).

A substantial body of evidence comprising observational studies, randomized controlled trials (RCTs), and meta-analyses, together suggest that physical activity has a substantial beneficial influence on cognitive function, and in particular executive function (Liu-Ambrose et al., 2010; Lowe et al., 2014). A meta-analysis of 18 exercise intervention studies conducted with older adults (aged 55–80) between 1966 and 2001, established that fitness training brings about robust benefits to cognition (Colcombe and Kramer, 2003). Exercisers experienced greater improvements (*g* = 0.478, *SE* = 0.029) in their performance on cognitive function tasks compared to control group participants (*g* = 0.164, *SE* = 0.028). The largest fitness based benefits were associated with improvements in executive processing (*g* = 0.68, *SE* = 0.052). This was a larger effect than was seen for other cognitive processes, namely processes measured using controlled (*g* = 0.461, *SE* = 0.035), spatial (*g* = 0.426, *SE* = 0.062), and speed tasks (*g* = 0.274, *SE* = 0.050). Type of fitness training appeared to influence the magnitude of benefit to cognitive function, with combined strength and aerobic training regimes producing larger effects (*g* = 0.59, *SE* = 0.049) than aerobic training alone (*g* = 0.41, *SE* = 0.037).

More recently a meta-analysis of 29 RCTs examining the effect of aerobic exercise training on neurocognitive performance was conducted. This analysis, including findings from large-scale randomized studies, demonstrated that increases in aerobic exercise were associated with modest but consistent improvements in executive function [*g* = 0.123, (95% CI: 0.021–0.225)] (Smith et al., 2010). This finding suggests markedly weaker effects compared to the meta-analysis of Colcombe and Kramer (2003). Several of the studies reviewed by Smith et al. (2010) included younger adults (e.g., 18–54) than those included in the meta-analysis of Colcombe and Kramer (2003). However, amongst the studies reviewed the age of participants was unrelated to improvements in executive function suggesting that the difference in effect size observed between the two meta-analyses is unlikely to be due to differences in the age composition of the participants included. Interestingly, and in contrast to the finding of Colcombe and Kramer (2003), effects did not differ between studies that included only aerobic exercise and those that included combined exercise programmes. Regards to the quality of the included studies, there were no differences in the effect of exercise on neurocognitive performance based on whether assessors were blinded or not, or whether intention to treat analysis was used or not.

Functional brain imaging studies have shed some light on the brain areas that may mediate the impact of physical activity on executive function (Hillman et al., 2008). When coupled with an RCT design such studies represent a particularly powerful approach to identifying cardiovascular training induced changes in brain function. For instance, a 6-month period of aerobic exercise has been shown to lead to enhanced performance on an attentional control task and greater activation of task-related frontal and parietal brain areas (Colcombe et al., 2004). A recent review of the literature has verified that physical activity appears to be most consistently linked to more efficient patterns of brain activity in frontal and parietal areas during tasks gauging executive function (Voelcker-Rehage and Niemann, 2013).

In addition to the findings from the meta-analyses of experimental studies outlined above, several longitudinal studies have also demonstrated that high levels of physical activity can attenuate declines in cognition (Sofi et al., 2011). In their meta-analysis of 15 prospective studies, Sofi et al. (2011) found that those who performed physical activity at baseline had a significantly reduced risk of cognitive decline during follow-up. Specifically, those who reported a high level of physical activity had a 38% reduced risk of cognitive decline relative to those who reported being sedentary (Hazard Ratio = 0.62, 95% CI: 0.54–0.70). This protective effect was similarly observed for those engaging in low to moderate levels of activity. Funnel plots of effect size vs. standard error suggested no evidence of publication bias. Although this meta-analysis did not discriminate between executive and non-executive measures several additional longitudinal studies point to a potential beneficial influence of physical activity on executive function (e.g., Barnes et al., 2003; Weuve et al., 2004).

Thus, the existing evidence from a broad range of studies shows that physical activity can lead to changes in executive function. An initial aim of the current study was therefore to verify that physical activity can attenuate age-related decline in executive function in a large sample of older English adults. A second aim was to examine dynamic changes in activity levels and executive function to rule out the role of unobserved stable confounders in explaining any association between physical activity and executive function. Specifically, fixed effects multilevel regression analyses were employed to statistically control for the role of non-observed time-invariant characteristics (e.g., sex, birth weight, education, genetic makeup, personality traits) (Allison, 2005). For instance, it is possible that non-observed factors like common genetic variation or childhood factors such as early adversity may underlie both adulthood physical activity and executive function and explain why these variables are interrelated (e.g., Gow et al., 2012). By examining within-person variation such non-observed time-invariant (i.e., factors that do not vary over time, specifically within the time-period of the study) confounders are statistically ruled out.

In contrast to the large number of studies investigating the beneficial effects of physical activity on executive function, very few studies have looked at the opposite possibility, i.e., that executive function facilitates future engagement in physical activity. The current study aimed to examine this possibility using longitudinal analyses in order to establish whether executive function may contribute to beneficial changes in physical activity over time.

### Executive function as a determinant of physical activity

Participation in physical activity typically requires individuals to effortfully overcome short term costs in return for long term gains. For example, when beginning a new exercise regime, short term costs such as inconvenience and discomfort are initially far more salient than long term (but not immediately apparent) benefits like improved fitness, weight loss and health (Hall and Fong, 2007). Consequently, it has been argued that efficient executive function is essential for both the adoption and maintenance of physical activity (Hall and Fong, 2007; Marteau and Hall, 2013). Executive function can be subdivided in different ways, but key components include volition, planning, purposive action, performance monitoring and inhibition (Lezak, 1995; Stuss and Levine, 2002). The necessity of each of these components for initiating physical activity is readily apparent. To successfully adopt or change physical activity behavior individuals must form a conscious intention about what activity to adopt (volition), identify the sequence of actions required to achieve the intended activity (planning), initiate and maintain focus on the chosen activity over time (purposive action), compare actual progress with planned progress over time, identify and correct mistakes (performance monitoring) and overcome the temptation to remain sedentary (inhibition).

In line with this, research suggests individuals who perform poorly on tests of executive function are less likely to enact physical activity intentions (Hall et al., 2008a,b) and to adhere to regular exercise classes (McAuley et al., 2011). Poor performance on executive function tasks has also been shown to prospectively predict low levels of physical activity and elevated body mass index (BMI) in children (Guxens et al., 2009; Riggs et al., 2010). In a recent study of older women (aged 65–75) changes in executive function over the course of a year where participants engaged in a resistance exercise intervention were positively associated with subsequent exercise adherence over the following year (Best et al., 2014). Whilst this evidence is suggestive of a role of executive function in contributing to physical activity, large scale nationally representative prospective studies have not yet provided evidence that efficient executive function is advantageous in the control of activity behavior, allowing individuals to engage in more physical activity over time relative to others.

### The current study

The present study investigates the relationship between executive functioning and physical activity measured at four time points over a 6-year period, using data from the English Longitudinal Study of Aging (ELSA) (Marmot et al., 2012).

This study aims to:

1. Replicate the established association between physical activity and executive function in a large sample of older English adults.
2. Improve on the analytic strategies used in previous studies by statistically controlling for stable non-observed factors (e.g., genetic factors, birth weight, education, early adversity, etc.) which may underlie both adulthood physical activity and cognitive function and explain why these variables are interrelated.
3. Test whether physical activity level predicts subsequent changes in executive function.
4. Test whether the efficiency of cognitive function at one point in time can be used to predict future engagement in physical activity.

## Methods

### Participants

The ELSA is a multi-wave longitudinal study of health and quality of life in a large sample of adults aged 50 years or older living in England. The initial ELSA sample was drawn from participants aged 50+ years who took part in the 1998, 1999, and 2000 waves of the Health Survey for England which was designed to represent the English adult population. The first wave of data collection was conducted in 2002 and subsequent waves were carried out every 2 years using both face-to-face interviews/tests and self-completion questionnaires. The sample selection, study design, and measures are described elsewhere (Marmot et al., 2003). Ethical approval for all the ELSA waves was granted from the National Research and Ethics Committee, and all procedures adhered to the Helsinki Declaration. Participants provided informed consent prior to study participation. Data on executive function and physical activity participation were collected in each of the four waves of the study examined as were details of participants' age, gender, education, wealth, and the presence of long-standing illness, as shown in Table 1. The analyses in the present study utilized 18,220 observations from 4555 participants who took part in all four study waves.

**Table 1:** **Descriptive statistics for each wave of the English Longitudinal Study of Aging examined**.

**Wave (*N*) Parameter**	**Wave 1 (*N* = 4555) M (SD)/%**	**Wave 2 (*N* = 4555) M (SD)/%**	**Wave 3 (*N* = 4555) M (SD)/%**	**Wave 4 (*N* = 4555) M (SD)/%**
Physical activity^a^	3.01 (0.75)	3.03 (0.72)	2.97 (0.75)	2.91 (0.80)
Executive function^b^	10.75 (3.10)	10.59 (3.14)	10.54 (3.22)	10.52 (3.35)
Age	62.34 (9.44)	64.66 (9.46)	66.5 (9.46)	68.62 (9.64)
Female	56.73%	56.73%	56.73%	56.73%
Education^c^	4.33 (2.26)	4.33 (2.26)	4.33 (2.26)	4.33 (2.26)
Log wealth	5.12 (0.66)	5.24 (0.63)	5.28 (0.61)	5.28 (0.63)
Long-standing illness	50.98%	53.17%	53.08%	53.74%

a *Frequency of engagement in mild/moderate/vigorous activity, ranging from 1 = hardly ever, or never to 4 = more than once a week*.

b *Composite score based on verbal fluency and letter cancelation task*.

c *Highest educational qualification, ranging from 1 = no qualification equivalent to 7 = degree or equivalent*.

### Measures

#### Physical activity

The physical activity questions were introduced as follows: “*We would like to know the type and amount of physical activity involved in your daily life*.” Participants were then asked three separate questions which gauged how frequently they engaged in sports or activities of (1) mild, (2) moderate, and (3) vigorous intensity. A card was then presented to participants detailing a broad set of typical daily physical activities considered mild (e.g., vacuuming, home repairs), moderate (e.g., walking at a moderate pace, gardening), and vigorous (e.g., running, cycling). The extent to which participants engaged in each type of physical activity was gauged using response options capturing activity levels on a monthly basis coded as follows: 1 = hardly ever, or never; 2 = one to three times a month; 3 = once a week; and 4 = more than once a week. Responses on the three items (mild, moderate, and vigorous activities) were combined to create a composite variable reflecting total physical activity participation. This variable was then standardized so that high scores equate to higher levels of activity, and regression coefficients could be easily interpreted.

As self-reported measures of physical activity may be affected by misreporting or social desirability biases we test whether the ELSA measure correlates with an objective indicator of physical functioning. As part of the ELSA study the time taken for participants to walk a distance of 8 feet at their regular or usual pace was recorded at each wave. The timed walk was completed with the use of a walking aid where necessary (approximately 4% of participants). The timed walk was repeated and the average of the two assessments was calculated. Across 11,711 observations on the study sample the observed correlation between self-reported physical activity and walking speed was 0.42 suggesting a moderate degree of correspondence in the current sample.

#### Executive function

In each wave participants completed two brief tests of executive function—a verbal fluency task and a letter cancelation task. The verbal fluency task used was a standard, well normed semantic category fluency test (Lezak, 1995; Tombaugh et al., 1999) where participants were asked to generate as many exemplars of a reference category (“animals”) as possible in 60 s. Verbal fluency tests, in particular letter fluency tests, are routinely used to assess executive dysfunction (Parker and Crawford, 1992; Phillips, 1997; Henry and Crawford, 2004). Numerous studies have demonstrated that damage to brain regions associated with EF is associated with poor performance on verbal fluency tests (e.g., Baldo and Shimamura, 1998; Schwartz and Baldo, 2001). Patients with frontal lobe damage/executive dysfunction reliably show large deficits in verbal fluency task performance, and importantly, these patients show differential deficits in verbal fluency tasks relative to more general cognitive tests of IQ and processing speed (Henry and Crawford, 2004).

As in the current study, verbal fluency tasks typically present participants with an unusual, unpracticed task (generating words based on specified orthographic criteria), specifying a goal which must be met without giving a strategy for doing so. Performance on verbal fluency tasks requires the successful operation of multiple specific EF processes, including working memory updating (keeping track of responses, keeping the target letter and rules in mind; Henry and Crawford, 2004), self-monitoring of performance, inhibition (of previously given or inappropriate responses) and strategic switching between “clusters” of responses (Hirshorn and Thompson-Schill, 2006). Performance on this animal naming task specifically reflects flexible, goal directed searching of semantic memory, performance monitoring and inhibition of previously named/inappropriate exemplars, and is sensitive to both aging and the beneficial effects of physical activity (Lindwall et al., 2012).

The letter cancelation task asked participants to locate and cross out as many occurrences of the letters P and W as they could on a page printed with 65 randomly ordered letters of the alphabet within 60 s. Letter cancelation tasks assess a key component of executive functioning, selective attention or the ability to focus on relevant stimuli while simultaneously ignoring or screening out irrelevant stimuli (Jurado and Rosselli, 2007; Diamond, 2013). Cancelation tasks also gauge sustained attention, visual search ability and mental speed and have been shown to have high reliability and validity (Uttl and Pilkenton-Taylor, 2001).

The ELSA index of executive function is based on the verbal fluency and letter cancelation tasks. These two tasks form three scales which are summed to produce a composite executive function score (Institute of Fiscal Studies, 2012) ranging from 0 to 20 derived as follows: (1) The number of animals named form a scale from 0 (0–7 animals named) to 8 (30+ animals named), (2) The number of letters reached in the letter cancelation task are recoded into a second scale ranging from 0 (0–174 letters reached) to 7 (450+ letters reached), and (3) The number of target letters missed by the participant in the letter cancelation task is recoded into a scale ranging from 0 (9 or more missed) to 5 (0–1 missed). Executive function index scores were standardized (mean = 0, SD = 1) so that regression coefficients could be interpreted as indicative of change in SD's of a unit change in the independent variable.

#### Covariates

Details of each participant's age in years, sex, highest educational qualification, non-pension wealth, and whether they had been diagnosed with a long-term illness were used for descriptive purposes and as control variables in the statistical models. The education variable was coded on a seven-point scale ranging from 1 = no qualification to 7 = degree or equivalent. The wealth measure captured a broad set of wealth sources including the value of housing, current and savings account balances, premium bonds, shares, and private debt (e.g., credit card debt and outstanding loans). This variable was log-transformed to reduce skew. The presence of long-standing illness was reported at each wave (present/not present).

### Statistical analysis

Multi-level random coefficient analyses were used to account for the hierarchical structure of the data, whereby non-independent repeated observations across the five waves (Level 1) were nested within participants (Level 2). Our analytic strategy was as follows. First we examined the cross-sectional association between physical activity and executive functioning for individuals (*i*) across the four waves examined (*t*). We used multilevel modeling and adjusted for participant age, wealth and the presence of long-standing illness at each wave, as well as unchanging characteristics sex and highest educational qualification. Dummy variables for each wave were included in the analyses (**Model 1**). Using standard notation these regression models can be summarized as follows:

**Model 1:** Level 1: Executive function_*ti*_ = β_0i_ + β_1i_(Physical activity_*ti*_) + β_2i_(Age_*ti*_) + β_3i_(Wealth_*ti*_) + β_4i_(Long-standing illness_*ti*_) + β_5_(Wave_*t*_) + *r*_*ti*_
Level 2: β_0*i*_ = γ_00_ + γ_01_(Gender_*i*_) + γ_02_(Education_*i*_) + *u*_0*i*_

Next we examined how changes in physical activity relate to changes in executive functioning by conducting a fixed effects analysis (**Model 2**). This analysis exploits the longitudinal nature of the data to test whether within-person variation in physical activity predicts within-person variation in executive functioning. By examining within-person variation such non-observed time-invariant (i.e., factors such as sex, birth weight, education, genetics, etc. that do not vary over time, specifically within the time-period of the study) confounders are essentially ruled out. The fixed effects analyses adjusted for changes in wealth and health that could explain any association between within-person changes in executive functioning and corresponding changes in physical activity. For example, the difference between a person's executive function level in each participating wave and the person's average level of executive function is represented by “Executive function_*ti*_ − Executive function_*i*_.” This fixed effects multilevel regression analysis eliminates unobservable individual heterogeneity (α) that remains stable across waves, thus producing an account of the executive function–physical activity relation that is free from the influence of important non-observed stable individual factors like genetic variation or experiences of childhood adversity.

**Model 2:** Level 1: Executive function_*ti*_ − Executive function_i_ = β_1i_(Physical activity_*ti*_ − Physical activity_*i*_) + β_2i_ (Age_*ti*_ − Age_*i*_) + β_3i_(Wealth_*ti*_ − Wealth_*i*_) + β_4i_(Long-standing illness_*ti*_ − Long standing illness_*i*_) + β_5_(Wave_*t*_) + (α_*i*_ − α_*i*_) + (*r*_*ti*_ − *r*_*i*_)

Finally, having identified whether the relationship between executive function and physical activity remains after adjustment for time-invariant factors we tested the direction of this relationship. Using multi-level modeling we investigated whether physical activity in a given wave (*t*) predicts executive functioning levels in the subsequent wave (*t* + 1) adjusting for the participant's executive functioning score and age at baseline (*t*) along with gender, education, wealth and health controls (**Model 3**). In **Model 4** longitudinal change in physical activity (from *t* to *t* + 1) is predicted by executive functioning at baseline (*t*).

**Model 3:** Level 1: Executive function_*t* + *1i*_ = β_0i_ + β_1i_(Physical activity_*ti*_) + β_2i_(Executivefunction_*ti*_) + β_3i_(Age_*ti*_) + β_4i_(Wealth_*ti*_) + β_5i_(Long-standing illness_*ti*_) + β_6_(Wave_*t*_) + *r*_*ti*_
Level 2: β_0_*i* = γ_00_ + γ_01_(Gender_*i*_) + γ_02_(Education_*i*_) + *u*_0*i*_

**Model 4:** Level 1: Physical activity_*t* + *1i*_ = β_0i_ + β_1i_(Executive function_*ti*_) + β_2i_(Physical activity_*ti*_) + β_3i_(Age_*ti*_) + β_4i_(Wealth_*ti*_) + β_5i_(Long-standing illness_*ti*_) + β_6_(Wave_*t*_) + *r*_*ti*_
Level 2: β_0*i*_ = γ_00_ + γ_01_(Gender_*i*_) + γ_02_(Education_*i*_) + *u*_0*i*_

## Results

The characteristics of the sample for each of the four waves of ELSA utilized in the study are detailed in Table 1. The average age of the sample at baseline was 62.34 years (SD = 9.44) and participants were mainly female (56.7%). Approximately 51% of the sample reported having been diagnosed with a long-standing illness at baseline.

### Cross-sectional relationship between physical activity and executive function

There was little variation in average levels of physical activity across waves (Min = 2.91, Max = 3.03), as shown in Table 1. Executive function scores also varied little across waves, with participants scoring from 10.52 to 10.75 out of 20 on average across the four waves. The correlation between physical activity and executive function appeared to strengthen as participants aged (Overall: *r* = 0.23, *p* < 0.001; Wave 1: *r* = 0.17, *p* < 0.001; Wave 2: *r* = 0.20, *p* < 0.001; Wave 3: *r* = 0.25, *p* < 0.001; Wave 4: *r* = 0.30, *p* < 0.001). Our first multilevel model (Table 2) showed that higher levels of physical activity were associated with better executive functioning (*B* = 0.05, *SE* = 0.01; *t* = 8.52, *p* < 0.001) after adjusting for age, gender, education, wealth, and health status. Our analyses indicates that a 1 SD increase in physical activity corresponded with a 0.05 SD increase in executive function scores.

**Table 2:** **The physical activity and executive function variables were standardized at the wave rather than the full sample level which is more appropriate**.

**Parameter**	**Executive function^a^**		**Executive function^a^**	
**Model**	**Random effects**		**Fixed effects**	
	**B (SE)**	***t***	**B (SE)**	***t***
Intercept	2.12 (0.12)	18.33^**^	1.31 (0.14)	9.23^**^
Physical activity^b^	0.05 (0.01)	8.52^**^	0.03 (0.01)	4.49^**^
Age	−0.03 (0.00)	−27.63^**^	−0.02 (0.01)	−1.87
Female	0.21 (0.02)	9.69^**^	–	–
Education^c^	0.11 (0.01)	20.63^**^	–	–
Log wealth	0.08 (0.01)	5.82^**^	0.03 (0.02)	1.51
Long-standing illness	0.00 (0.01)	−0.34	0.02 (0.01)	1.64
Wave 1^d^	−0.13 (0.01)	−8.58^**^	−0.03 (0.06)	−0.52
Wave 2^d^	−0.11 (0.01)	−8.70^**^	−0.05 (0.04)	−1.28
Wave 3^d^	−0.07 (0.01)	−5.50^**^	−0.03 (0.02)	−1.36

a *Composite score based on verbal fluency and letter cancelation task*.

b *Frequency of engagement in mild/moderate/vigorous activity, ranging from 1 = hardly ever, or never to 4 = more than once a week*.

c *Highest educational qualification, ranging from 1 = no qualification equivalent to 7 = degree or equivalent*.

d *Base category for analysis of Wave effects is Wave 4*.

^*^p < 0.01,

** *p < 0.001*.

### Fixed effects model of the physical activity–executive function link

Our fixed effects model showed that within-person changes in executive functioning were associated with within-person changes in physical activity levels (*B* = 0.03, *SE* = 0.01; *t* = 4.49, *p* < 0.001). Thus, the relationship between physical activity and executive functioning appeared to be robust to strict statistical control for person-level time-invariant factors. However, approximately 40% of the relationship between physical activity and executive functioning could be attributed to unobserved time-invariant confounding factors. Our fixed effects analyses suggested that an increase of 1 SD in physical activity was associated with approximately a 0.03 SD increase in executive function amongst older adults.

### Longitudinal models of the direction of the physical activity-executive function link

Next, we investigated the direction of the physical activity-executive function relationship using longitudinal random-effects models. We tested the impact of physical activity on subsequent executive functioning adjusting for age, gender, education, wealth, long-standing illness and executive function at baseline. This analysis showed that physical activity is linked to an increase in executive function over time *B* = 0.03, *SE* = 0.01; *t* = 5.09, *p* < 0.001), as shown in Table 3.

**Table 3:** **The physical activity and executive function variables were standardized at the wave rather than the full sample level which is more appropriate**.

**Parameter**	**Executive function (*t* + 1)**		**Physical Activity (*t* + 1)**	
	**B (SE)**	***t***	**B (SE)**	***t***
Intercept (*t*)	1.06 (0.08)	13.74^**^	0.37 (0.09)	4.24^**^
Physical activity (*t*)^a^	0.03 (0.01)	5.09^**^	0.49 (0.01)	65.02^**^
Executive function (*t*)^b^	0.59 (0.01)	84.71^**^	0.05 (0.01)	6.54^**^
Age (*t*)	−0.02 (0.00)	−23.18^**^	−0.01 (0.00)	−14.85^**^
Female (*t*)	0.09 (0.01)	6.91^**^	−0.06 (0.01)	−4.42^**^
Education (*t*)^c^	0.04 (0.00)	13.75^**^	0.02 (0.00)	5.64^**^
Log wealth (*t*)	0.03 (0.01)	3.33^**^	0.10 (0.01)	8.93^**^
Long-standing illness (*t*)	−0.05 (0.01)	−4.01^**^	−0.16 (0.01)	−11.60^**^
Wave 1^d^	−0.08 (0.02)	−5.36^**^	0.09 (0.02)	5.36^**^
Wave 2^d^	−0.03 (0.01)	−2.20^*^	0.02 (0.02)	1.26

a *Frequency of engagement in mild/moderate/vigorous activity, ranging from 1 = hardly ever, or never to 4 = more than once a week*.

b *Composite score based on verbal fluency and letter cancelation task*.

c *Highest educational qualification, ranging from 1 = no qualification equivalent to 7 = degree or equivalent*.

d *Base category for analysis of Wave effects is Wave 3*.

* p < 0.01,

** *p < 0.001*.

In the opposite direction we found that high levels of executive function predicted a longitudinal increase in physical activity levels (*B* = 0.05, *SE* = 0.01; *t* = 6.54, *p* < 0.001), while adjusting for age, gender, education, wealth, long-standing illness and physical activity at baseline. A 1 SD increase in executive function was linked to a 0.05 increase in physical activity over time. This increase was found to be equivalent in magnitude to a 2.5 point improvement in education (measured on a seven-point scale ranging from 1 = no qualification to 7 = degree or equivalent) or moving from pass secondary level education to holding a degree. Furthermore, the magnitude of the longitudinal change in physical activity associated with elevated executive function levels was 55% greater than the increase in executive function associated with high levels of physical activity.

## Discussion

In a study of more than 4500 adults aged 50+ physical activity and executive function were closely interlinked. This association remained after controlling for demographic and health characteristics. Furthermore, in strictly controlled fixed effects analyses we demonstrated that dynamic within-person changes in executive function corresponded with parallel changes in physical activity. Critically, our analyses showed that the magnitude of the relationship between physical activity and neurocognitive performance appeared to be strongest in the direction from executive function to physical activity. Previously, strong executive abilities have been found to prospectively predict high levels of physical activity in children (Riggs et al., 2010; Pentz and Riggs, 2013) and exercise adherence in older adults (McAuley et al., 2011). The current study suggests that executive abilities may have favorable effects on activity levels at the population level in older adults. This is in line with temporal self-regulation theory (Hall and Fong, 2007) which proposes that pre-potent response inhibition (a key facet of executive functioning) will be essential for the enactment of behaviors like physical activity which require short term effort for long term health gain.

We found evidence that the relationship between physical activity and executive function is bidirectional. Those with poor executive function showed subsequent decreases in their rates of participation in physical activity and older adults who engaged in sports and other activities involving physical exertion tended to retain high levels of executive function over time. This research strengthens and extends existing evidence demonstrating that physical activity can buffer the effects of aging on cognitive decline, particularly in relation to the executive functions (Agrigoroaei and Lachman, 2011; Erickson et al., 2012; Weinstein et al., 2012). This bi-directionality is encouraging from a behavior change standpoint as it suggests that interventions which promote either physical activity or more efficient executive function may have the capacity to produce reciprocal benefits.

Physical activity has been successfully increased among older adults using strategies such as telephone counseling and group-based programmes (Wilcox et al., 2006); pedometer-driven interventions (Talbot et al., 2003); and education-based interventions (van der Bij et al., 2002). Although challenging and labor intensive, there is evidence that executive function can also be improved through training. Lasting improvements in the executive functions have been achieved in older adults using cognitive training techniques and mindfulness meditation (Ball et al., 2002; Willis et al., 2006). As the largest beneficial effects of these cognitive interventions are typically seen in those with the lowest levels of executive function and strong deleterious impulsive tendencies (Hofmann et al., 2012), it may be possible to target interventions at those with the greatest capacity to benefit. However, the extent to which such interventions can enhance engagement in physical activity and whether this could attenuate the declines in physical functioning associated with poor executive functioning (Watson et al., 2010; Koehler et al., 2011) remains to be identified.

Our study has several strengths. Firstly, this is the first study to explicitly investigate the bidirectional nature of the executive function–physical activity relationship over time. The finding that there is a reciprocal, mutually beneficial relationship between the two is of theoretical and practical importance, aiding the interpretation of cross-sectional studies and highlighting new avenues for the design of interventions to facilitate healthy aging. Secondly, the large sample provided precise estimates and high statistical power. Thirdly, our estimates were independent of factors (e.g., wealth, chronic illness) that are known to independently influence engagement in physical activity.

Finally, the use of fixed effects regression techniques allowed us to determine that the executive function–physical activity association is robust to non-observed stable confounding variables. This analysis showed that such non-observed variables explained 40% of the association between executive function and physical activity identified in the standard multilevel regression model. The fixed effects regression results point to the need for non-experimental studies examining the link between executive function and physical activity to either measure or econometrically account for stable potential confounding variables including childhood adversity, genetic endowment and personality traits. Failure to consider these often unobserved variables could lead to inflated estimates of the association between physical activity and executive function. This is because such non-observed variables could lead to changes in both activity levels and executive function and explain why these variables are interrelated.

Fixed effects models using panel or sibling/twin data can appropriately account for childhood adversity or differences in genetic makeup which cannot change. However, fixed effects models cannot fully account for the potential confounding role of personality traits which, although highly stable, have been shown to respond to maturation and life events (e.g., Boyce et al., in press). It may be more appropriate to measure individual differences in personality traits in order to rule out confounding resulting from these variables.

In terms of limitations, the fixed effects regression cannot rule out third variables which are time-varying and not included in the regression model. For instance, if marked changes in health behavior (e.g., increased cigarette or high calorie food consumption) occurred between waves this could negatively impact on both executive function and physical activity levels. Similarly, pronounced changes in health over time may not be adequately captured by the control for chronic conditions included in the analyses. These and other unobserved time-varying variables could account for the association between changes in executive function and physical activity. It is important that future research utilize experimental designs to rule out this potential confounding and to identify whether changes in executive function can causally affect physical activity.

Furthermore, the measure of physical activity used in the ELSA study was participant reported and may have been prone to response bias (e.g., social desirability effects, differential use of response scales). Prior research has shown that the association between objectively recorded and self-reported physical activity is typically modest with several studies showing that both methods share approximately 25% of common variance (e.g., Fahrenberg, 1996; Welk et al., 2004; Nagels et al., 2007). The presence of measurement error is therefore likely to have attenuated the link between executive function and physical activity in the current study (e.g., Celis-Morales et al., 2012).

In the current study we identified a moderate positive association (*r* = 0.42, *p* < 0.001) between the measure of physical activity utilized and a measure of participant walking speed demonstrating that this self-report measure correlates with objective differences in physical functioning. Furthermore, by including analyses of within-person changes in activity levels we can account for individual differences in social desirability bias and in the use of self-report response scales. This is because we examine changes in response so a systematic tendency to over- or underestimate physical activity will be statistically removed. Future research capitalizing on the recent inclusion of accelerometry for the assessment of physical activity in population representative longitudinal studies (e.g., Griffiths et al., 2013) would help verify or challenge the pattern of results identified in the current study.

Due to the constraints of a large-scale multipurpose study such as ELSA the present analyses utilized brief measures of executive functioning (verbal fluency/letter cancelation). While well normed, objective tests, it must be noted that the executive functions are multi-faceted, and comprehensive measurement of executive abilities requires a full neuropsychological test battery. Brief measures such as those utilized in the current study are likely to be less reliable and this measurement error may lead to a downward bias in the size of the effects observed. Similarly, the tasks used will inevitably reflect an element of general cognitive function in addition to “purely” executive functioning, particularly in terms of processing speed. Future studies would ideally explore a broader range of executive abilities to investigate whether particular facets of executive functioning (e.g., planning, sustained attention, self-monitoring) are equally predictive of physical activity.

Finally, whilst our study has shown that relatively strong executive abilities can lead to improvements in activity levels, we were unable to examine the mechanism underlying this change in the current study. Previous research points to the role of the executive functions in allowing intentions to engage in physical activity to be implemented, potentially by inhibiting distractions and facilitating the enactment of behavior (Hall and Fong, 2007). This represents a promising direction for further research (Allan et al., 2010, 2011).

In conclusion, in this large longitudinal study of older English adults we have demonstrated a mutually beneficial, reciprocal relationship between physical activity and executive function that cannot be attributed to either observed or non-observed confounders. Our study suggests that in old age the relationship between executive function and physical activity is dynamic such that changes in executive function can enhance and promote physical activity over time and that changes in activity level can improve future executive function. This finding points to the potentially reciprocal benefits of intervention strategies which aim to concurrently promote executive function and physical activity in older adults.

## Funding

Michael Daly gratefully acknowledges funding support from the Economic and Social Research Council (ES/L010437/1). David McMinn was funded by the Scottish Government, Rural and Environment Science & Analytical Services (RESAS) division. The funders had no role in study design, data collection and analysis, interpolation of these data, decision to publish, or preparation of the manuscript.

### Conflict of interest statement

The authors declare that the research was conducted in the absence of any commercial or financial relationships that could be construed as a potential conflict of interest.
